# Targeting Tumor Hypoxia with Nanoparticle-Based Therapies: Challenges, Opportunities, and Clinical Implications

**DOI:** 10.3390/ph17101389

**Published:** 2024-10-18

**Authors:** Sujit Kumar Debnath, Monalisha Debnath, Arnab Ghosh, Rohit Srivastava, Abdelwahab Omri

**Affiliations:** 1 NanoBios Lab, Department of Biosciences and Bioengineering, Indian Institute of Technology Bombay, Mumbai 400076, India; drskdeb@gmail.com (S.K.D.); monalishadeb19@gmail.com (M.D.);; 2 Department of Chemistry and Biochemistry, The Novel Drug and Vaccine Delivery Systems Facility, Laurentian University, Sudbury, ON P3E 2C6, Canada

**Keywords:** hypoxia, tumor biology, cancer therapy, nanoparticle delivery, therapy resistance

## Abstract

Hypoxia is a crucial factor in tumor biology, affecting various solid tumors to different extents. Its influence spans both early and advanced stages of cancer, altering cellular functions and promoting resistance to therapy. Hypoxia reduces the effectiveness of radiotherapy, chemotherapy, and immunotherapy, making it a target for improving therapeutic outcomes. Despite extensive research, gaps persist, necessitating the exploration of new chemical and pharmacological interventions to modulate hypoxia-related pathways. This review discusses the complex pathways involved in hypoxia and the associated pharmacotherapies, highlighting the limitations of current treatments. It emphasizes the potential of nanoparticle-based platforms for delivering anti-hypoxic agents, particularly oxygen (O_2_), to the tumor microenvironment. Combining anti-hypoxic drugs with conventional cancer therapies shows promise in enhancing remission rates. The intricate relationship between hypoxia and tumor progression necessitates novel therapeutic strategies. Nanoparticle-based delivery systems can significantly improve cancer treatment efficacy by targeting hypoxia-associated pathways. The synergistic effects of combined therapies underscore the importance of multimodal approaches in overcoming hypoxia-mediated resistance. Continued research and innovation in this area hold great potential for advancing cancer therapy and improving patient outcomes.

## 1. Introduction

Cancer has become a global public concern due to the aging of the global population, complex treatments, and massive economic burden. Chemotherapy is the most preferable approach for cancer alone or in conjunction with other treatment options like immunotherapy, photodynamic therapy, photothermal therapy, radiotherapy, etc. [1]. Radiation therapy is an integral part of cancer therapy, and the radiosensitivity of tumor tissues depends on the tissue O_2_ tension. This is why radiologists primarily focus on the distribution of oxygen partial pressure in tumor tissues. Hypoxia represents a significant physiological stressor characterized by an inadequate oxygen supply to specific regions or organs, surpassing their regular demand within the tissue microenvironment. This phenomenon is particularly prevalent in cancerous tissues, notably solid tumors. Under normal circumstances, tissue oxygen tension typically ranges between 24 and 66 mmHg. However, this tension plummets drastically to below 10 mmHg in hypoxic conditions. Oxygen plays a crucial role in stabilizing deoxyribonucleic acid (DNA)-damaging free radicals that are induced by radiation. Cancer cells exhibit heightened resistance to radiation therapy in its absence, necessitating higher doses than usual for effective destruction. Moreover, hypoxia diminishes the sensitivity of cancer cells to chemotherapy and immunotherapy during treatment [2]. Notably, the presence of hypoxia within the tumor microenvironment not only fosters metastasis but also confers resistance to reactive oxygen species (ROS)-generated cancer therapies [3]. On the other hand, hypoxia-inducible factor-1 alpha (HIF-1α) promotes vascular endothelial growth factor (VEGF) production through the HIF-1α signaling pathway, resulting in the proliferation of blood vessels. However, this elevated blood circulation cannot fulfill the requirement for a progressed tumor. In such a situation, the tumor cell sustains due to the transformation of the glucose metabolism pathway, which is, again, upregulated by the HIF-1α pathway [4].

Numerous studies have demonstrated the intervention of bio-carriers and nanoparticle-based carriers in increasing the oxygen concentration in the tumor microenvironment. Likewise, it improves drug delivery to the tumor side and the efficacy of radiotherapy [5]. In addition, they also proliferate the adaptive and innate antitumor immune cells to enhance the efficacy of immunotherapy. Understanding hypoxia and the mediated pathways is essential for a better understanding of drug targeting through the nanocarrier system to the tumor microenvironment. This review comprehensively examines various hypoxia-mediated pathways and potential interventions aimed at mitigating hypoxia-associated challenges. A thorough classification of drugs is presented to elucidate their direct or indirect action against hypoxia. Furthermore, this review sheds light on the limitations of current treatments involving anti-hypoxic drugs, nanoparticulate-based formulations, and their prospects for targeted delivery in combating hypoxia within the context of cancer therapy.

## 2. Intervention of Hypoxia-Mediated Pathways and Cancer Therapy

Hypoxia exerts profound effects on the pathobiology of tumor growth, influencing various cellular processes that are crucial for cancer progression. These effects include alterations in cell proliferation, tumor tissue survival in adverse microenvironments, the ability to breach migration barriers, the evasion of immune system surveillance, and dissemination to distant organs. Consequently, targeting hypoxia-mediated pathways has emerged as a promising therapeutic strategy for cancer.

### 2.1. Hypoxia-Inducible Factor-1 and Targeted Therapy

Hypoxia-inducible factor-1 (HIF) is the critical mediator responsible for hypoxic responses, comprising heterodimeric factors that are further categorized as HIF-1, HIF-2, and HIF-3. In normoxic conditions, proline residues on the α-subunit of HIF-1 undergo hydroxylation by prolyl hydroxylases (PHDs), leading to ubiquitination and subsequent degradation via the von Hippel–Lindau protein (pVHL) and the 26S-dependent proteasome. However, in hypoxic conditions, HIF-1α inhibits PHD activity, resulting in its stabilization and phosphorylation. Subsequently, HIF-1α forms a dimer with HIF-1β, binds to hypoxia-responsive elements (HREs), and modulates the expression of target genes. While HIF-1α expression is typically upregulated, HIF-2α expression is downregulated in tumor tissues [6].

HIF-1α exhibits homology with HIF-2α and HIF-3α and is implicated in various cellular processes. HIF-2α, which is predominantly expressed in adult vascular endothelial cells, lungs, the heart, and placenta during embryonic development, plays a crucial role in regulating the expression of genes such as erythropoietin (EPO), glucose transport proteins (GLUT-1 and GLUT-3), and vascular endothelial growth factor (VEGF), thereby influencing angiogenesis and metabolic adaptation. Moreover, HIF-2α is involved in upregulating the transcriptional activity and expression of HIF-3α and stimulates tumor progression through the macrophage lactate/HIF-2α/ATP6vod2 cascade [7,8].

**Figure 1:**
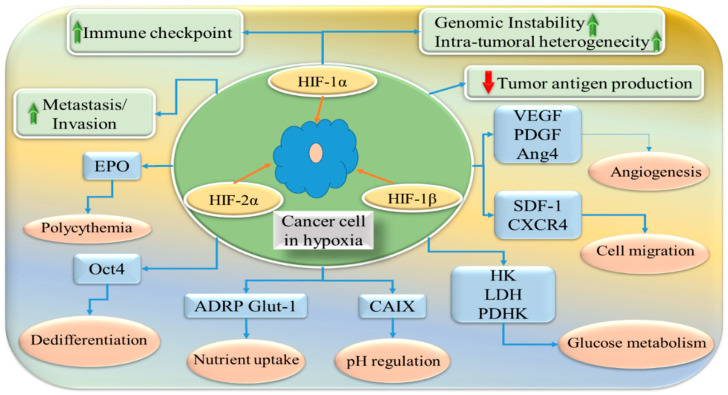
Hypoxia-inducing factor-mediated signaling pathways. HIF-1α, HIF-2α, and HIF-1β are the most prominent factors that cause hypoxia. Several genes are overexpressed in hypoxia, resulting in different proteins, growth factors, and enzyme malfunction. These changes trigger heterogeneity, such as nutritional deficiency, angiogenesis, metabolic changes, acidosis, and immunosuppression. VEGF—vascular endothelial growth factor; PDGF—platelet-derived growth factor; Ang4—angiogenin-4; SDF-1—stromal cell-derived factor; CXCR4—CXC chemokine receptor type 4; HK—hexokinase; LDH—lactate dehydrogenase; PDHK—pyruvate dehydrogenase kinase; CA-IX—carbonic anhydrase-IX; ADRP Glut-1—adipose differentiation-related protein glucose transporter-1; Oct4—octamer-binding transcription factor 4; EPO—erythropoietin.

The adaptive response to oxygen mediated by HIF-1 promotes various tumorigenic processes, including nutrient deficiency, angiogenesis, metabolic changes, acidosis, and immunosuppression. HIFs induce the expression of TWIST (Twist-related protein), a transcription factor regulating epithelial–mesenchymal transition (EMT), and activate the Sma- and Mad-related (SMAD) pathway, which is crucial for EMT and metastasis. Furthermore, HIF-1 stimulates cancer cell extravasation by inhibiting vascular endothelial cell adhesion through the induction of angiopoietin-related protein 4 (ANGPTL4) activity. More than 40 gene transcriptions are stimulated during hypoxia (Figure 1) [7].

Targeting HIF-1α represents a promising approach to overcome hypoxia-induced drug resistance in cancer. Studies have explored direct gene silencing and indirect inhibition via signaling pathways such as PI3K/Akt/mTOR. Additionally, the inhibition of heat shock protein 90 (HSP90), which is involved in HIF-1 transcription, has shown efficacy in bladder cancer treatment [9].

Hypoxia triggers malignancy and metastasis in solid tumors by promoting genetic mutations in key regulators such as Bcl-2 and p53. Moreover, hypoxia induces the transcription of genes that are crucial for tumor survival and progression, including vascular endothelial growth factor (VEGF), carbonic anhydrase-9 (CA IX), and glucose transport proteins (GLUT-1 and GLUT-3). CD73, which is regulated by HIF-1α, emerges as a promising target for suppressing cancer progression [10].

In certain instances, hypoxia induction during anticancer therapies has been explored to trigger vascular repair processes, suppressing the homology-directed repair of damaged DNA. Alternative approaches involve suppressing oxygen consumption using mitochondrial electron transport chain inhibitors to mitigate hypoxia-associated challenges. Additionally, angiogenic therapy with VEGF has shown promise in enhancing vasculogenesis and tissue remodeling in ischemic tissues, providing a potential avenue for hypoxia management in cancer therapy [11].

### 2.2. Metabolic Reprogramming by Hypoxia and Its Targets

Metabolic reprogramming is a hallmark feature of hypoxia and its inducers. Hypoxia sensitizes the tumor cells to adapt to metabolic stress and facilitates metabolism by inducing several intra- and inter-metabolic steps (Table 1). Different metabolic pathways are activated to survive and proliferate in low-oxygen environments. Thus, addressing hypoxia-induced tumor metabolism is a viable method in cancer treatment.

### 2.3. Hypoxia-Mediated Adaptation to Apoptosis and Its Therapeutic Target

Hypoxia exhibits dual roles in either promoting or preventing cell death, which is contingent upon the cancer type and experimental conditions. Reversing the adaptation of cell death signals that are induced by hypoxia holds significance for targeting hypoxic tumors [16]. Both natural and synthetic medications targeting early or late pathways, including proteasomal inhibitors, protein kinase inhibitors, and cytotoxic agents, are currently under investigation and demonstrating efficacy in sensitizing cancer cells [17].

### 2.4. Genomic Instability and Its Target

Genomic instability arises from defective DNA repair mechanisms, a hallmark characteristic of cancer. Hypoxia is linked to impaired DNA repair, exacerbating the sensitivity of cancer cells to various DNA-damaging chemotherapeutic agents, such as poly (ADP-ribose) polymerase inhibitors and mitomycin C [18]. Notably, inhibiting hypoxia-induced DNA damage repair presents a promising new approach for targeting the most aggressive components of solid tumors.

### 2.5. Angiogenesis and Possible Targets

New blood vessels often exhibit aberrant malignancy characteristics, including immaturity and leakiness. Hypoxia stimulates angiogenesis, perpetuating the hypoxic environment and establishing a vicious cycle. Normalizing the tumor vasculature, which restores aberrant blood vessels, can reduce tumor invasion and metastasis while enhancing the efficacy of immunotherapy, radiation therapy, and chemotherapy. Notably, immune therapy-induced normalization of blood vessels facilitates the infiltration of immune effector cells, underscoring the potential benefits of co-administering antiangiogenic agents with immunotherapy [19].

Antiangiogenic therapy, which was first introduced in 1971 for cancer treatment, aims to inhibit the formation of new blood vessels [20]. Bevacizumab, an antiangiogenic drug, blocks the binding of vascular endothelial growth factor (VEGF) to its receptors. Other antiangiogenic agents, such as lenvatinib (a multi-kinase inhibitor of the VEGF receptor) and cabozantinib (a tyrosine kinase inhibitor), are also recommended in cancer therapy. Although long-term antiangiogenic treatment can lead to tumor shrinkage, it may also induce the development of a more aggressive and metastatic phenotype [21].

### 2.6. Inhibition of PI3K/AKT/mTOR Pathway

The PI3K/AKT/mTOR pathway plays a significant role in regulating the expression of HIF-1α, a well-established fact in the scientific literature. Consequently, extensive research efforts have focused on downregulating PI3K expression as a promising strategy. The inhibition of the PI3K/AKT/mTOR signal transduction pathway has proven effective in reducing HIF-1α expression. For instance, Dactolisib (BEZ_235_) negatively influences the mTOR/AKT/PI3K pathways [22].

Furthermore, dual inhibitors like PKI-587 have been developed to simultaneously target the mTOR/AKT/PI3K signal cascade and the DNA damage-repair (DDR) pathway. PKI-587 has demonstrated efficacy in enhancing the radiosensitization of hepatocellular cells, highlighting its potential in combination therapies. Indeed, combining radiotherapy with anti-hypoxic therapy has shown promise in eliminating cancerous cells [23].

## 3. Classification of Anti-Hypoxic Drugs

Different classes of drugs can be designated as anti-hypoxic drugs. They work either directly or indirectly to treat hypoxic conditions.

### 3.1. Hypoxia Inhibitor

Lowering the production and enhancing the degradation of hypoxia-inducible factor-1 (HIF-1) are two pivotal targets for mitigating its effects on tumor progression, metastasis, and therapy resistance. The reduction of HIF-1 production can be achieved through two main approaches: transcription and translation inhibitors. Transcription inhibitors target the activation of HIF-1 by the transcription coactivator p300. Medications like Chetomin destabilize the CH1 domain of p300, thus impeding its interaction with HIF-1. Similarly, drugs such as Bortezomib interfere with the interaction between HIF-1 and p300. Echinomycin, an antimicrobial cyclic peptide, binds to the recognition sequence of HIF-1, reducing its transcriptional activity and inducing cell death in glioma and breast cancer cell lines [24].

The PI3K/Akt/mTOR signaling pathway is known to increase HIF-1 levels primarily by elevating the rate of HIF-1 protein translation, enabling cancer cells to stabilize HIF-1 even under normal oxygen conditions. Translation inhibitors play a crucial role in downregulating HIF-1. Drugs like EZN-2968, topotecan, and 2-methoxy estradiol inhibit HIF-1 translation and nuclear translocation [25].

**Figure 2:**
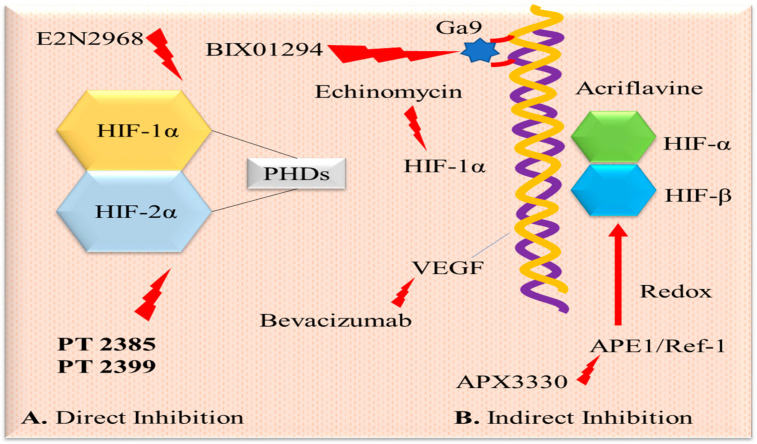
Pharmacological inactivation of HIF signaling. (**A**) PT2399 and PT2385 are potent and selective HIF-2α antagonists that directly bind to HIF-2α and inhibit it. E2N2968 inhibits HIF-1α via direct binding. PHD is prolyl hydroxylase domain enzyme. (**B**) Indirect inhibition is through various pathways by inhibiting transcription, translation, HIFα mRNA expression, HIFα protein synthesis, protein stabilization, accumulation, etc.

Histone methylation has emerged as another critical factor in the malignant progression of tumors under hypoxic conditions [26]. Certain substances affect the stability and degradation of the HIF-1 protein. The molecular chaperone Hsp90 is essential for HIF-1 stability, and pharmacologic inhibitors like geldanamycin increase HIF-1 ubiquitination and proteasomal degradation in renal cell carcinoma cell lines, independent of VHL [27]. Some inhibitors target HIF-1α or HIF-2α directly, while others indirectly modulate upstream or downstream signaling cascades associated with HIF (see Figure 2).

### 3.2. Hypoxia-Activated Prodrugs

Another special category is hypoxia-activated prodrugs (HAPs), which are bioreductive drugs that are activated in hypoxic conditions and can actively target the hypoxic regions of solid tumors [28]. These are inert compounds undergoing enzymatic reduction to become pharmacologically active within the tumor microenvironment (TME), particularly in hypoxic conditions. Cellular oxidoreductases trigger the reductive metabolism of these drugs, which include nitro groups (e.g., nitroimidazoles), quinones (e.g., mitomycin C), aromatic N-oxides (e.g., tirapazamine (TPZ)), aliphatic N-oxides (e.g., AQ4N), and transition metal complexes (e.g., cobalt (III) and copper (II) complexes for lethal chemical ligand release) [29].

Numerous hypoxia-activated prodrugs are currently undergoing clinical trials at various stages. These prodrugs exhibit reversible cytotoxicity under normoxic conditions, acting as mono/difunctional DNA alkylators, intra- and inter-strand crosslinkers, DNA strand breakers, or inhibitors of specific enzymes that are involved in DNA replication and repair, such as topoisomerase II by TPZ [30]. Notably, topoisomerase inhibitors have been shown to inhibit HIF activity indirectly.

Hypoxia-activated prodrugs have been extensively studied for their ability to target hypoxic tumor cells. For instance, topotecan, a topoisomerase-I inhibitor, is successfully used as a second-line treatment for small-cell lung and ovarian cancers [31]. However, despite the promising clinical potential of tirapazamine and evofosfamide as HAPs, these drugs failed to obtain regulatory approval after phase III clinical trials.

### 3.3. Agents for Molecular Targeting

Hypoxia affects several molecular pathways, and pinpointing these specific molecules has paved the way for targeting the hypoxic pathways responsible for hypoxia-mediated resistance to anticancer therapies. For instance, doxorubicin/daunorubicin prevents the binding of HIF-1 to the hypoxia-responsive element (HRE) sequence [25]. Antisense HIF-1α treatment inhibits HIF-1α expression through antisense gene transfer, resulting in the downregulation of VEGF translation and reduced tumor micro-vessel density.

Other compounds targeting hypoxia-related pathways include PX-478 (a HIF-1α inhibitor), which suppresses Foxp3 and VEGF transcription and/or expression. POM-1 (an ENTPD2 inhibitor) depletes myeloid-derived suppressor cells (MDSCs) and impedes tumor development [32]. Anti-carbonic anhydrase IX (CAIX) antibodies inhibit HIF-1 and topoisomerase-1 by targeting CAIX, rendering CAIX+ tumor cells more vulnerable to immune system destruction. SLC-0111 (a CAIX inhibitor) blocks tumor cell glycolysis and tumor microenvironment (TME) acidification. SCH58261, an A2AR antagonist, also blocks adenosine’s immunosuppressive effects.

Furthermore, benzopyranyl 1,2,3-triazole has been identified as an HIF-1 inhibitor due to its promotion of HIF-1 hydroxylation. This compound also dose-dependently downregulates VEGF expression [7].

### 3.4. Supplemental Oxygenation

Combining immunotherapy with oxygen supplementation appears promising and advantageous for reducing tumor hypoxia and accumulating extracellular adenosine driven by HIF-1-CD39/CD73. This approach weakens the A2AR/A2BR-mediated pleiotropic immunosuppressive cascade in the hypoxic tumor microenvironment (TME) [33]. Perfluorocarbons (PFCs) are synthetic hydrocarbons in which hydrogen atoms are substituted by fluorine atoms, rendering them highly stable and capable of dissolving large quantities of oxygen. PFC-based oxygen carriers show potential for repurposing as oxygenation agents to combat hypoxia in the TME. They can effectively load and unload oxygen at a rate that is even twice as fast as red blood cells (RBCs) [34]. To enhance biocompatibility, PFCs are either encapsulated or emulsified. The first-generation PFCs, perftoran and fluosol, underwent successful and safe clinical trials involving over 2000 patients [35]. However, it was noted that they faced limitations in penetrating thin blood vessels. Second-generation PFCs were developed with reduced particle sizes of less than 200 nm to facilitate their entry into narrow blood vessels for improved oxygen delivery.

### 3.5. Recombinant Anaerobic Bacteria

**Figure 3:**
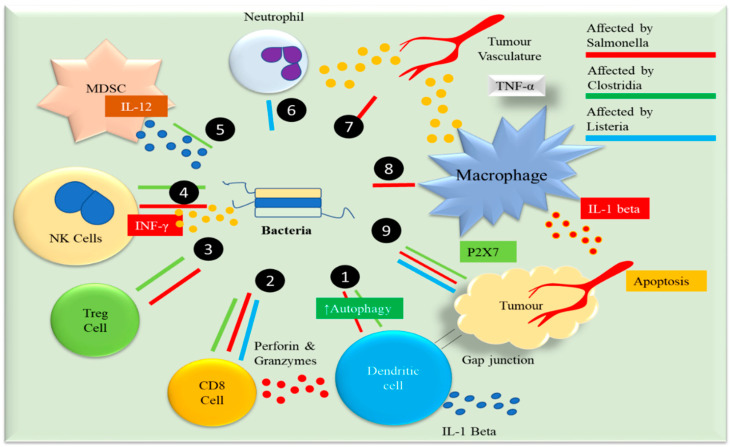
Recombinant anaerobic bacteria: 1. Autophagy: *Salmonella typhimurium*, *Listeria monocytogenes*, and *Clostridium difficile* toxins. 2. When dendritic cells are exposed to tumor antigens and interact with bacterial components, they produce high amounts of IL-1, a proinflammatory cytokine that activates CD8+ T cells. 3. By activating TLR5 on activated CD8+ T cells, bacterial flagellin augments active CD8+ T cells’ antitumor response. Perforin and granzyme proteins that are secreted by activated CD8+ T lymphocytes efficiently kill tumor cells in primary and metastatic cancers. 4. Additionally, both flagellin and TLR5 signaling decrease the amount of CD4+CD25+ regulatory T (Treg) cells, boosting the antitumor response of activated CD8+ T cells. 5. The flagellin produced by *S. typhimurium* stimulates the production of interferon- (IFN-), a crucial cytokine in innate and adaptive immunity. 6. MDSCs infected with Listeria have an immune-stimulatory phenotype characterized by increased IL-12 production, augmenting CD8+ T and NK cell responses. 7. Both *S. typhimurium* and *Clostridium* infections have the potential to cause a significant rise in neutrophils 8. When macrophages contact bacterial components (LPS and flagellin) or cancer cells infected with *Salmonella*, the macrophage inflammasome is activated, boosting the release of IL-1 and TNF into the tumor microenvironment. 9. Increased neutrophil production of TNF and TNF-related apoptosis-inducing ligand (TRAIL) enhances the immune response and induces apoptosis in tumor cells.

Tumor-targeting bacteria employ various mechanisms to combat cancer [36]. These bacteria prefer thriving and proliferating within tumors, stimulating anticancer immune responses. Bacteria can be engineered to produce and disseminate anticancer drugs tailored to therapeutic requirements through genetic modification or synthetic bioengineering. The main purpose of this genetic engineering is to enhance antitumor effects and concurrently decrease bacterial pathogenicity. Utilizing live tumor-targeting bacteria alone or combined with other anticancer agents shows promise for enhancing clinical outcomes. These bacteria are vectors that promote gene expression by delivering monoclonal antibodies like genetically engineered T cells, PD-L1, PD-1, and CLTA-4. In addition, they were used to deliver other materials, like cytotoxic proteins (FlaB, FasL, and ClyA), cytokines (CCL21, IL-8, LIGHT, and IL-2), anti-angiogenic factors (thrombospondin 1 and endostatin), and pro-apoptotic activity (TNF-related ligands like Noxa, Fas ligand, and TRAIL), and prodrug-converting enzymes (cytosine deaminase) [37]. Figure 3 describes the mechanism of action of recombinant anaerobic bacteria-mediated tumor inhibition. Although these facultative and obligate anaerobic bacteria can infiltrate hypoxic regions to suppress their growth rate, the major challenge is to avoid damaging the normal tissues. In a study, *Salmonella typhimurium* strain SL7207 was engineered to place an essential gene under hypoxic conditions in such a manner that it could survive in anaerobic conditions without disturbing its functions [38]. The strain was observed to be a safe bacterial vector for antitumor therapies without compromising its other functions. In a recent study, NIR-nanoantenna-sensitized engineering bacteria was developed to ablate hypoxic tumors effectively and dual selectively. An engineered *E. coli* MG1655 surface was coated with lanthanide upconversion nanoparticles, enabling the NIR laser-switchable generation/secretion of hemolysin E perforin to kill cancer cells [39]. This NIR-responsive nanoantenna-sensitized switching in engineering bacteria is an alternative therapeutic option against hypoxia tumors.

### 3.6. Mitochondrial Electron Transport Chain Inhibitors

Utilizing vascular-targeting agents can further enhance the targeting of clostridia to solid tumors, enabling the treatment of tiny tumors that have yet to develop necrotic regions. Among these agents are DMXAA (5,6-dimethylxanthenone-4-acetic acid), which primarily functions by increasing tumor necrosis factor production in tumors, and the tubulin-binding agent combretastatin 4A along with its derivative ZD 6126 [40]. These medications induce the rapid and selective blockade of tumor blood vessels within 16–24 h of administration, leading to necrosis.

### 3.7. Inhibitors of the Mitochondrial Electron Transport Chain

Recent findings indicate that reducing oxygen consumption through inhibiting the mitochondrial electron transport chain can alleviate hypoxia within the tumor microenvironment. Notably, papaverine, an FDA-approved antispasmodic drug, and its derivatives effectively reduce mitochondrial oxygen consumption by inhibiting complex I of the electron transport chain [29]. Another promising approach involves using atovaquone-loaded human serum albumin nanoparticles, which have successfully alleviated hypoxia within the tumor microenvironment, along with an anti-PD-1 antibody [41].

### 3.8. Hypoxia-Targeted Immunotherapy

Incorporating anti-HIF drugs into immunotherapy presents a suitable strategy for mitigating cancer cell resistance. Within a hypoxic environment, the activities of immunosuppressive cells, particularly regulatory T cells (Tregs) and myeloid-derived suppressor cells (MDSCs), re subdued. Consequently, the cytotoxic function of T cells is diminished. In contrast, the expression of Tregs, energy, inhibitory receptor programmed death-1 (PD-1), extracellular remodeling, differentiation, function, and the recruitment of MDSCs to the tumor site is augmented [42]. Studies have illustrated that HIF-1α promotes the upregulation of adenosine-generating enzymes such as CD39/CD73 [43]. This enzyme leads to extracellular adenosine accumulation, which binds with cyclic adenosine monophosphate (cAMP), thereby enhancing the expression of A2A/A2B adenosine receptors on tumor-reactive T cells. Consequently, T-cell effector functions are suppressed by initiating protein kinase A-mediated signaling, resulting in immunosuppression. An alternative approach involves restoring the physiological oxygen levels in the tumor microenvironment (TME) and enhancing immunotherapy by combining oxygenation therapy with A2A adenosine receptor antagonists. Oxygenation therapy aids in diminishing the activity of HIF-1α through oxygen-dependent degradation. Conversely, A2A adenosine receptor antagonists impede downstream adenosinergic signal transduction and prevent immunosuppressive transcription. The inhibition of CTLA-4 and PD-1, approved by the Food and Drug Administration (FDA), is one of the most promising immunotherapy approaches [44].

### 3.9. Small Interfering RNA-Mediated Gene Silencing

One highly promising approach in cancer treatment involves suppressing the expression of the oncogene responsible for tumor growth. Numerous techniques have been utilized to downregulate target genes, with small interfering RNA (siRNA)-mediated gene silencing emerging as particularly potent. Extensive evidence underscores the effectiveness of nanocarrier-mediated siRNA delivery in silencing the expressions of key oncogenes such as HIF-1α and CD73 [10].

## 4. Drawback of Current Treatment with Anti-Hypoxic Drugs

Despite numerous developments in anti-hypoxic drugs in cancer therapy, there are a few drawbacks and limitations that need to be reviewed to explore the potential application of these drugs.

### 4.1. Assessment of In Vivo Tumor Hypoxia

#### 4.1.1. Lack of Validated Hypoxia Diagnostic Modalities

Developing a practical and accurate method for detecting hypoxia is crucial for the success of hypoxia-targeted therapies. A comprehensive clinical approach is necessary to diagnose tumor hypoxia, particularly for identifying individuals who would benefit from targeted treatment. While endogenous hypoxia indicators show promise for monitoring the administration of hypoxia-activated prodrugs (HAPs) to hypoxic tissues, their widespread implementation is hindered by the lack of proven imaging or molecular indicators that meet regulatory standards.

#### 4.1.2. Limitations of Positron Emission Tomography Scan

Positron emission tomography (PET), a potential tool for monitoring tumor hypoxia, provides surrogate signals for areas of poor oxygenation and serves as a proxy for hypoxia-mediated alterations in vivo. However, various factors can compromise its accuracy, including tumor type-specific constraints [45]. While PET tracers combined with nitroimidazole-based triggers can offer some specificity, they cannot provide absolute pO_2_ readings independently [46]. Moreover, this challenge is exacerbated in metastatic tumors, where PET signals may vary significantly among different neoplastic masses within the same patient.

### 4.2. Challenges in Hypoxia-Activated Prodrug Delivery

#### 4.2.1. Tumor Interstitial Fluid Pressure

Although hypoxia-activated prodrugs (HAPs) present a promising strategy for targeting hypoxic regions within tumors, their effectiveness relies on adequate delivery to the tumor site, a factor that is often underestimated in the quest for precision in drug development. The material characteristics of tissue structure, including the physical microarchitecture comprising stromal and extracellular matrix (ECM) components, along with heightened tumor interstitial fluid pressure (TIFP), play crucial roles in tumor invasion [47].

#### 4.2.2. The Disruption of the Vasculature

TIFP significantly contributes to the repeated disruption of the tumor vasculature, exacerbating intra-tumor hypoxia. However, TIFP poses a significant obstacle to the dispersion of HAPs. Additionally, it affects the efficacy of chemical probes that are intended to detect the hypoxic environment within tumors [48].

#### 4.2.3. The pH of the Microenvironment

Tumor acidosis, a microenvironmental factor associated with hypoxia, can hinder the efficacy of drugs by upregulating multiple HIF1 targets. Acidification happens when hypoxia-activated prodrugs (HAPs) enter the extracellular environment, which is caused by the protonation of basic amino groups. This acidic milieu significantly impacts the absorption of drugs by cancer cells. However, HAPs with nitrogen mustard alkylating capability may be an exception, as they tend to be more stable in acidic conditions and are more likely to accumulate in malignant cells [49].

### 4.3. Hypersensitivity Towards HAPs

Patients may develop hypersensitivity to hypoxia-activated prodrugs (HAPs) following standard-of-care therapy. Studies suggest that HAPs may be more effective in tumors exhibiting significant hypoxia, as indicated by their mode of action and imaging assessments. Sensitizing tumors to HAPs can be achieved by administering other clinically approved medications known to induce temporary hypoxia. Bevacizumab (Avastin) is one such option for combination therapy. When combined with other anti-VEGF/antiangiogenic drugs, bevacizumab targets the surviving neoplastic fraction. For instance, dichloroacetate (DCA), a pyruvate dehydrogenase kinase (PDK) inhibitor, reactivates mitochondrial respiration, further reducing oxygen levels [49,50].

### 4.4. Compensatory Upregulation

When one subunit of the hypoxia-inducible factor (HIF), either HIF1 or HIF2, is inhibited, the other subunit often undergoes compensatory upregulation. Targeting HIF1 and HIF2 signaling simultaneously could lead to more efficient tumor elimination. 2-Methoxyestradiol (2-ME2), a naturally occurring metabolite of estradiol, presents a promising option for the dual targeting of HIF1 and HIF2 receptors. Research has shown that 2-ME2 impedes the nuclear translocation of both HIF1 and HIF2 proteins, leading to the decreased expression of their downstream targets, such as VEGF, lactate dehydrogenase A, and cyclin D1. Furthermore, in hepatocellular carcinoma (HCC) cell lines, 2-ME2 has been found to enhance the efficacy of sorafenib, a kinase inhibitor, particularly under hypoxic conditions. This synergistic effect of sorafenib and 2-ME2 reduced proliferation and increased apoptosis in vitro and in vivo. In the context of bevacizumab-resistant tumors, VEGF inhibition has been shown to activate an HIF-driven pathway, contributing to persistent tumor growth. Evidence suggests that while VEGF inhibition initially affects tumor size, it may also lead to an increased metastatic burden and the shedding of circulating tumor cells (CTCs), primarily due to heightened intra-tumor hypoxia [51].

**Figure 4:**
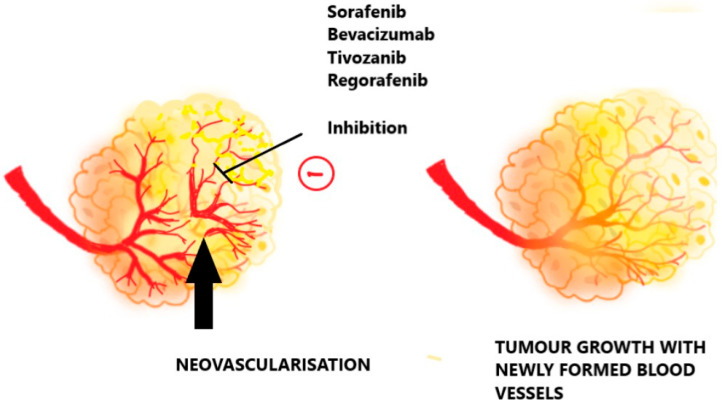
Combinatorial models of targeting tumor hypoxia.

Given the potential for tumor cells to switch between HIF isoforms, targeting HIF1 and HIF2 is likely more effective in anticancer therapy. Understanding the resistance mechanisms will facilitate the development of suitable combinations of targeted therapies, including HIF, NF-κB, STAT3, and CA9/12 inhibitors, which could be activated in response to low oxygen levels [52]. Hypoxia-activated prodrugs (HAPs) may also play a crucial role in delaying or overcoming resistance in cancers that initially respond to angiogenesis inhibitors but later re-emerge in a more hypoxic environment, as demonstrated in animal studies. The combinatorial approach to targeting tumor hypoxia is illustrated in Figure 4.

Focusing on both HIF1 and HIF2 will likely be more successful because tumor cells may flip between isoforms, and inhibiting both isoforms will likely be more successful as an anticancer therapy. Understanding the resistance mechanisms will enable us to test suitable combinations of targeted medications (HIF, NF-B, STAT3, and CA9/12 inhibitors) in conjunction with this new class of therapies that are activated when the patient is subjected to low oxygen levels [52]. As proven in animal studies, HAPs may be especially beneficial in delaying or overcoming resistance in cancers that react initially to angiogenesis inhibitors but eventually re-emerge in a more hypoxic environment.

### 4.5. Targeting Specific Compartments

In experimental therapeutic models, mTORC1 inhibitors appear to exert a more pronounced effect on the oxygenated regions of the brain, suggesting reduced efficacy in hypoxic compartments. The relationship between mTORC1 and hypoxia in patients’ tumors is likely to be more complex, with limited studies available on this topic [53]. Consequently, combining hypoxia-activated prodrugs (HAPs) with mTORC1 inhibitors may offer a strategy to target both less hypoxic and profoundly hypoxic regions within tumors.

### 4.6. Challenges of a 3D Microsystem

Hypoxia affects all cellular components of a tumor, including cancer and stromal cells, promoting an immunosuppressive microenvironment characterized by the infiltration of various immune cells. Biomimetic tumor platforms, facilitated by advanced three-dimensional (3D) systems, enable the integration of diverse cell types from the tumor microenvironment, aiding in the prediction of effective drug treatments for highly hypoxic and aggressive tumors [54].

### 4.7. Toxicities

HAPs like TPZ and its derivatives, which are activated at intermediate oxygen tension levels within tumors, may effectively target the relevant fraction of tumor cells exhibiting radiation resistance. However, these drugs carry the risk of activating in normal tissues, leading to dose-limiting side effects. Clinical trials combining TPZ with cisplatin and/or radiation did not demonstrate additional benefits within the appropriate dose range [55]. Dose-limiting toxicities pose a significant challenge in the development of hypoxia-activated prodrugs, prompting interest in the exploration of novel and less toxic compounds. Additionally, activated prodrugs may have a bystander effect by diffusing active cytotoxic molecules from hypoxic to normoxic tumor areas. Prototype 2-nitroimidazole-conjugated HAPs like bromoisophosphoramide TH-302 and dinitrobenzamide PR-104 faced challenges with toxicity during phase I/II clinical trials [55].

## 5. Intervention of Nanoparticulate-Based Anti-Hypoxic Drug Delivery

Nanoparticles (NPs) smaller than 50 nm can penetrate cells easily, interact with biological systems inside and outside the cell surface, and play a role in various cancer therapies. Nanomedicines have emerged as promising solutions for pharmaceutical challenges such as low water solubility, poor bioavailability, and systemic toxicity. Despite numerous efforts, only a handful of nanoformulations have made it to the market, including Onivyde^®^, Doxil^®^ (liposomal formulation), Abraxane^®^ (albumin-bound paclitaxel), Myocet^®^ (liposomal formulation of DOX), and Daunoxome^®^ (liposomal daunorubicin) [56]. The variable normoxic tissues and hypoxic microenvironments allow for tumor-specific drug delivery by reducing the partial oxygen pressure. Hypoxia-induced modifications can serve as a basis for designing nanomedicines tailored to target the tumor microenvironment (TME). Several nanotherapeutics (refer to Table 2) are being explored to target TME hypoxia and overcome the limitations of current anti-hypoxic therapies. Stimuli-responsive nanoparticles that release anticancer drugs in response to intracellular redox potential and an acidic pH in the TME are under development. Furthermore, developing hypoxia-sensitive prodrugs enhances the efficacy of hypoxia-sensitive nanocarrier systems for controlled drug release at the tumor site.

### 5.1. Lipoidal Nanoparticles

Lipoidal nanoparticles are utilized effectively in drug delivery, transporting various therapeutic agents to infection sites. These nanoparticles boast several advantages, including being non-toxic, easy to manufacture, and highly biocompatible, making them a safer alternative to other nanocarrier systems [68]. Glioma, a prevalent type of malignant brain tumor known for its invasiveness and aggressiveness, poses significant challenges for surgical eradication or being cured. To address this, hypoxia-responsive lipid polymer nanoparticles have been developed for applications in photodynamic therapy, photothermal therapy, chemotherapy, and fluorescence-guided surgery [57]. These lipoidal nanoparticles are loaded with doxorubicin (DOX), indocyanine green (ICG), the glioma-targeting peptide angiopep-2, and poly(nitroimidazole)25 [P-(Nis)25]. The hydrophilic component of P-(Nis)25 exhibits hypoxia-responsive properties, enabling the encapsulation of DOX and ICG. Upon irradiation with an 808 nm laser, ICG induces a hypoxic environment, while DOX inhibits glioma growth.

An O_2_ self-sufficient liposomal formulation (LipoMB/CaO_2_) has been developed to enhance photodynamic therapy efficacy in hypoxic tumors [58]. This formulation incorporates calcium oxide (CaO_2_) and methylene blue (MB) into liposomes. The photosensitizer MB activates ^1^O_2_ and induces lipid peroxidation, degrading the liposome under short-time irradiation. MB also enhances the contact area of CaO_2_ with H_2_O, progressively increasing O_2_ production. Elevated O_2_ levels regulate the tumor’s hypoxic environment, reducing tumor hypoxia, suppressing tumor growth, and inhibiting tumor metastasis with fewer side effects.

To address the premature release of the radical promoter 2,2-azobis [2-(2-imidazoline-2-yl)propane] dihydrochloride (AIPH), lipid nanoparticles were loaded with AIPH and indocyanine green [59]. These nanoparticles melted at 44 °C under near-infrared light due to the photothermal effect, preventing premature drug leakage. Free radicals generated from AIPH exhibited anticancer effects in normoxic and hypoxic environments. Similarly, a self-enriched photodynamic therapy (PDT) approach was developed, where a photosensitizer is loaded into perfluorocarbon nanodroplets to overcome oxygen shortages in the tumor microenvironment [69]. Perfluorocarbon exhibits a longer ^1^O_2_ lifetime and higher O_2_ capacity, ensuring a rich oxygen supply for improved PDT efficiency in cancer therapy.

Many approaches are evolving to improve the impaired transportation of oxygen. LEAF-4L6715 is a liposomal nanocarrier of trans-crocetin (TC), which was injected to enhance the oxygenation of vascular tissue [62]. In vitro, this liposomal formulation increases the reoxygenation properties of free TC. Due to the liposomal formulation, the half-life of this drug was increased six-fold in healthy mice. This formulation is under a phase 2 clinical trial focusing on the treatment of preoperative hypo-fractionated radiotherapy in patients with localized or locally advanced soft tissue sarcoma [61].

### 5.2. Polymeric Nanoparticles

Polymeric nanoparticles offer numerous advantages, including protecting drugs and therapeutic molecules from the biological environment, controlled release properties, and an improved therapeutic index and bioavailability. Various polymers, such as poly(ɛ-caprolactone) (PCL), polylactic-co-glycolic acid (PLGA), polylactic acid (PLA), the Eudragit series, and polyethylene glycol (PEG), possess unique properties suitable for drug delivery applications [70]. Depending on specific requirements, these polymers can be utilized in reservoir systems (nanocapsules) or matrix systems (nanospheres).

Catalase (CAT), a catalytic enzyme required to decompose H_2_O_2_ into O_2_, is essential for reducing tumor hypoxia. However, CAT faces challenges, like a short half-life, protease-induced degradation, and immunogenicity. CAT was incorporated into PLGA nanoparticles to overcome these hurdles, effectively addressing these limitations [60]. When H_2_O_2_ penetrates the nanoparticles, the nanoparticle shell ruptures due to internal pressure, releasing CAT and evolving O_2_. Platinum, an anticancer drug, was incorporated into the same nanoformulation to demonstrate combined oxygen therapy and chemotherapy.

In the context of NK cell therapies for solid tumors, hypoxia-induced immunosuppression in the tumor microenvironment poses a challenge by hindering the cytotoxic activity of natural killer (NK) cells. Manganese dioxide nanoparticles (MnO_2_ NPs) were developed to degrade tumor-producing H_2_O_2_ [63]. Subsequently, PLGA was used to coat these MnO_2_ NPs to improve their biocompatibility and accelerate O_2_ production. These biodegradable PLGA-MnO_2_ NPs serve as oxygen-evolving nanomaterials for hypoxic tumor treatment. Upon penetration into cancerous spheroids, these nanoparticles downregulate HIF-1α expression and reduce hypoxia, offering an alternative strategy to regulate cytotoxic immune cells in the tumor microenvironment and improve adoptive NK cell therapy. The efficacy of PEG-, PLL-, and PLGA-based nanoparticles modified with transferrin and embedded with daunorubicin was evaluated on leukemia cells under hypoxic conditions [64]. These DNR-Tf-NPs demonstrate potential as ideal nanocomposites for enhancing drug sensitivity in tumor treatment under hypoxia.

### 5.3. Metal Nanoparticles

Metallic nanoparticles (MNPs) have garnered significant interest and are widely utilized in drug delivery applications. Surface modification or functionalization of metallic nanoparticles allows them to conjugate with various ligands and antibodies, making them effective drug carriers [71]. As previously discussed, perfluorocarbons (PFCs) are crucial in oxygen transportation. However, the release of oxygen often faces challenges due to low delivery efficiency. To address this issue, PFC-loaded hollow Bi_2_Se_3_ nanoparticles were developed to facilitate the timely supply of oxygen under near-infrared (NIR) light, thereby enhancing radiation therapy [65]. The application of NIR light triggers a burst release of oxygen from the nanoparticles, aiding in eradicating hypoxia-related radioresistance.

In addition, TaOx nanoparticles were conjugated with PFC nanodroplets for hypoxia treatment [66]. PEG was employed to stabilize this nanocomposite. Upon exposure to X-ray radiation, TaOx nanoparticles concentrate the radiation energy within tumor cells. Simultaneously, PFC serves as an oxygen reservoir, releasing oxygen to promote oxygenation. Gold nanoparticles (GNPs) have been extensively investigated for their application in photothermal cancer therapy. The potential of GNPs in radiotherapy (RT) was explored to overcome radioresistance in hypoxic conditions [67]. These GNPs feature a silica core with a gold shell. It was observed that GNPs enhanced the antitumor effect when combined with RT compared to RT alone.

## 6. Scope of Nanoparticle-Based Targeted Delivery of Anti-Hypoxic Drugs

In recent years, researchers have been tirelessly striving to address the challenges posed by the heterogeneous nature of tumors. Hypoxia, a prevalent characteristic observed in 50–60% of solid tumors, often leads to drug resistance through the hypoxia-induced adaptive response governed by the transcription regulation gene HIF-1α. The inability of most anticancer drugs to effectively penetrate the tumor microenvironment (TME) or maintain adequate concentrations therein increases susceptibility to drug resistance. Tumor cells undergo genetic alterations in response to the hypoxic environment, facilitating rapid adaptation and thwarting hypoxia-induced cell death [72].

Hypoxia-targeted strategies aim to enhance the oxygen concentration, convert active metabolites through enzymatic reduction from bio-reductive prodrugs, and develop small-molecule inhibitors targeting HIF-1α [56]. The efficacy of nano-based therapy incorporating HIF-1α siRNA holds promise in overcoming cancer drug resistance. Nanotechnology has ushered in a new era in cancer treatment by providing an improved platform for combination therapy. Targeted drug delivery can be achieved through direct and passive means.

Passive targeting entails accumulating a significant amount of drug at the tumor site due to enhanced vascular permeability and limited lymphatic drainage from the TME, known as the enhanced permeability and retention (EPR) effect. Active targeting, on the other hand, involves ligand-binding nanoparticles entering cells via receptor-mediated endocytosis. Polymeric nanocarriers ranging from 100 to 150 nm can prevent unwanted drug accumulation in organs like the liver, lungs, and spleen through the reticuloendothelial system (RES) [73].

Nanoparticle-based targeted delivery not only enhances the EPR effect of drugs but also prolongs their retention in the body by delaying renal clearance. Nanoparticles can be specifically designed to target features of the TME, such as hypoxia and acidosis, facilitating more precise drug release at the intended site. Several nanotechnology-based strategies have garnered attention from the scientific community, including tissue reoxygenation, the inhibition of HIF-1 activity, genetic silencing of HIF-1 expression, and the encapsulation of drugs that disrupt the HIF-1 signaling pathway [31].

Another innovative approach involves camouflaging membrane-coated nanoparticles to improve the delivery of anticancer drugs to hypoxic tumors. In a study, red blood cell-coated poly(lactic-co-glycolic acid) nanoparticles (RBC-PLGA-NPs) containing curcumin (a chemotherapeutic agent) and tirapazamine (a hypoxia-activated molecule) were prepared [74]. This combination effectively generated reactive oxygen species (ROS), activated caspases, and damaged the DNA of cancerous cells.

### 6.1. Lungs Targeted

Hypoxia triggers lung injury by activating lipid peroxidation and impairing cellular antioxidant defenses, leading to hypoxia-induced pulmonary hypertension. In treating hypoxic lung injury, intrathecal administration of liposomal-α tocopherol (LAT) has shown promise [75]. LAT was found to normalize the lung phospholipid composition and exert remarkable anti-hypoxic effects. Furthermore, studies have shown that topotecan, a topoisomerase I inhibitor, indirectly inhibits HIF signaling. This topotecan treatment can be a viable second-line option for small-cell lung cancer [31]. Additionally, nanoparticle fabrication using biocompatible polymers has demonstrated enhanced targeted drug delivery to the lungs despite the hypoxic microenvironment.

### 6.2. Liver Targeted

The PI3K/AKT/mTOR signaling pathway is recognized as a critical driver of hepatocellular carcinoma (HCC) activation. Dactolisib (BEZ235) acts as an inhibitor in this pathway, but its use alone can lead to serious side effects and lacks specificity. Utilizing a polymeric nanocarrier to deliver BEZ235 offers a promising alternative for anti-hypoxic therapy. In a liver-targeted approach, BEZ235-loaded nanoparticles containing anti-GPC3 were developed [22]. This nano-assembly significantly reduced HIF-1α accumulation and effectively eradicated hepatocellular carcinoma.

Additionally, a combination of radiotherapy and anti-hypoxic therapy has shown effectiveness in targeted HCC therapy [23]. This study combined radiation with PKI-587, a dual inhibitor of PI3K, targeting the mTOR pathway. Combinational therapy holds promise as a potential approach for liver cancer treatment. Vorinostat, a histone deacetylase inhibitor, has been found to disrupt HIF-1α protein accumulation [76]. Vorinostat demonstrated anti-hypoxic effects in liver cancer-derived cells by degrading HIF-1α subunits. Therefore, employing a nanoparticulate system loaded with vorinostat could offer a novel approach to targeting and treating liver cancer.

### 6.3. Colon Targeted

In colon cancer targeting, enhancing ROS-induced cytotoxicity presents another strategy to manage hypoxia in the tumor microenvironment. Intravenous administration of high-dose vitamin C (ascorbate) suppressed HIF-1α signaling while targeting hypoxia-induced colon cancer [29]. Directly targeting vitamin C to the colon using nanotechnology could reduce the required dose due to its more localized distribution. The anti-inflammatory flavonol quercetin has been identified as an inhibitor of HIF-1 transcriptional activity in colon cancer cell lines [76]. Under hypoxic conditions, quercetin induced apoptosis and inhibited AMPK, leading to a dose-dependent decrease in HIF-1α production. Thus, utilizing nanoparticulate-based targeted delivery of quercetin holds promise for improving treatment outcomes in colon cancer.

### 6.4. Breast Targeted

Hyperbaric oxygen therapy has been shown to mitigate late radiation toxicity in breast cancer patients effectively [77]. However, cisplatin, a potent anticancer drug effective against triple-negative breast cancer (TNBC), induces hypoxia, leading to cisplatin resistance in cancer stem cells. To address this challenge, combination therapy involving metformin and gefitinib was introduced to sensitize TNBC cells to cisplatin [78]. Gefitinib, an epidermal growth factor receptor (EGFR) inhibitor, and metformin, an AMP-activated protein kinase (AMPK) activator, were utilized in this study. Metformin served as an anti-hypoxic agent by destabilizing HIF-1α via inhibiting mTORC1 activity. Additionally, metformin reversed hypoxia-mediated resistance to gefitinib.

Photodynamic therapy (PDT) often induces hypoxia in the tumor microenvironment (TME). To enhance PDT efficacy, an oxygen nanocarrier was developed to target the TME [79]. In a preclinical study, a chlorine e6-encapsulated oxygen nanocarrier was tested on 4T1 murine breast cancer cells, demonstrating significant inhibition of HIF-1α activity by providing oxygen supply to the TME. In another approach, cycloheximide, an inhibitor of the HIF-1 protein, enhanced the activity of doxorubicin against the 4T1 breast cancer cell line [76]. These examples underscore the importance of targeted delivery to breast cancer, which can be achieved through nanotechnology intervention.

### 6.5. Brain Targeted

Bevacizumab, a well-known anti-VEGF drug, has shown efficacy against various cancers. However, it exhibits resistance against the U87 GBM cell line under hypoxic conditions [80]. Carbonic anhydrase-IX (CA-IX), a marker overexpressed during HIF-1α transcription under hypoxia in brain tumors, plays a crucial role in malignant brain tumor therapy via the downstream inhibition of HIF-1. Combining CA-IX inhibitors with bevacizumab has significantly reduced the growth rate of the U87 GBM cell line. The inhibitory activity of CA-IX enhances the anti-VEGF effect of bevacizumab.

Moreover, the expression of c-Mesenchymal epithelial transition (c-Met) protein is observed in the brain tumor microenvironment under hypoxia. Thus, inhibiting c-Met expression has emerged as a novel strategy against brain tumors in hypoxic environments. However, RNA interfaces have shown limited effectiveness in reaching the target site due to instability and inadequate delivery. To address this challenge, a cationic solid lipid nanoparticle delivery system for c-Met siRNA was developed for systemic glioblastoma treatment [81]. This study demonstrated increased accumulation of c-Met siRNA within brain tumors, leading to reduced tumor growth.

### 6.6. Pancreas Targeted

Hypoxia-activated prodrugs offer a novel strategy to target anoxic cells by either reoxidizing or undergoing cellular reduction via cellular reductases. In a phase II clinical trial, TH-302, a hypoxia progenitor, was evaluated for its efficacy [82]. Combining gemcitabine with TH-302 enhanced its cytotoxic activity against pancreatic cancer cells in hypoxic environments. Additionally, a study on epigallocatechin-3-gallate (EGCG), a bioactive flavonoid derived from green tea, demonstrated a dose-dependent reduction in HIF-1α proliferation and mRNA expression in the pancreatic cell line PANC-1 [83]. These findings underscore the potential benefits of nanoparticulate delivery of anti-hypoxic drugs in managing pancreatic cancer.

## 7. Conclusions

The review provides an in-depth analysis of the critical role of hypoxia in tumor progression and the innovative use of nanoparticles to address this challenge. Tumor hypoxia is a major factor contributing to cancer resistance against conventional therapies, necessitating the development of novel treatment strategies. This review meticulously highlights the potential of nanoparticle-based platforms to deliver therapeutic agents directly to the hypoxic tumor microenvironment. This targeted delivery not only enhances the efficacy of the treatment but also minimizes systemic side effects, making it a promising approach in cancer therapy.

The detailed examination of hypoxia-mediated pathways, including the pivotal role of hypoxia-inducible factors (HIFs), provides valuable insights into the mechanisms that drive hypoxia-induced resistance. The review identifies key therapeutic targets by elucidating these pathways and proposes nanoparticle-based strategies to counteract hypoxia’s adverse effects effectively. Integrating drug delivery systems, oxygen carriers, and gene therapy vectors into nanoparticle formulations represents a multifaceted approach with significant promise for improving therapeutic outcomes. The importance of multimodal strategies that combine various therapeutic modalities to enhance treatment efficacy and overcome the limitations of single-agent therapies is emphasized throughout the review.

Despite the promising potential of nanoparticle-based therapies, the review also addresses the challenges associated with their clinical translation. Regulatory hurdles, potential side effects, and the need for comprehensive clinical trials are critical considerations that must be addressed to ensure these therapies’ safe and effective implementation in clinical settings. Further research is called for to optimize nanoparticle formulations, refine delivery methods, and validate the efficacy and safety of these innovative treatments through rigorous clinical testing. By highlighting these challenges and proposing potential solutions, the review sets a clear agenda for future research in hypoxia-targeted cancer therapies.

In conclusion, this review provides an insightful overview of the advancements and challenges in nanoparticle-based therapies targeting tumor hypoxia. The detailed analysis of hypoxia pathways and the innovative therapeutic strategies proposed offer valuable guidance for researchers and clinicians seeking to develop more effective cancer treatments. This review significantly contributes to the ongoing efforts to improve cancer therapy and patient outcomes by addressing the critical issue of tumor hypoxia and exploring cutting-edge nanoparticle technologies.

### 7.1. Impact on Real-World Outcomes

The advances in nanoparticle-based therapies for targeting tumor hypoxia represent a significant breakthrough in cancer treatment. Tumor hypoxia is a critical barrier to the effectiveness of traditional therapies, contributing to resistance and poor patient outcomes. By utilizing nanoparticles, it is possible to deliver therapeutic agents directly to the hypoxic regions of tumors, enhancing drug efficacy and minimizing systemic toxicity. This targeted approach could revolutionize the current treatment guidelines, making therapies more effective and personalized.

One of the most immediate real-world impacts could be the improvement in treatment outcomes for patients with hypoxic tumors, which are notoriously difficult to treat with conventional methods. The precision of nanoparticle-based delivery systems means that higher concentrations of drugs can be directed to the tumor site, potentially reducing the necessary dosage and associated side effects. This type of delivery could lead to better patient compliance, fewer adverse reactions, and improved quality of life for cancer patients. Economically, nanoparticle-based therapies could initially increase healthcare costs due to the need for specialized manufacturing and delivery systems. However, the improved effectiveness and reduced need for prolonged treatments could result in significant cost savings over time. Additionally, overcoming drug resistance in tumors could reduce the need for multiple lines of therapy, further decreasing the overall treatment costs.

Despite these potential benefits, the adoption of nanoparticle-based therapies in clinical practice faces several hurdles. Regulatory challenges, the need for extensive clinical trials to establish safety and efficacy, and the high development and production costs are significant barriers. Furthermore, integrating these advanced therapies into existing treatment protocols requires substantial training for healthcare providers and adjustments in infrastructure. Addressing these challenges is crucial for successfully translating nanoparticle-based therapies from the laboratory to the clinic.

### 7.2. Key Areas for Improvement

While nanoparticle-based therapies offer promising solutions, there are several areas for improvement to maximize their potential. One major challenge is the optimization of nanoparticle formulations to enhance their stability, bioavailability, and targeting efficiency. Researchers need to develop nanoparticles that can navigate the complex tumor microenvironment, penetrate deeply into hypoxic regions, and release their payloads in a controlled manner. Another critical area is the improvement of imaging and monitoring techniques to track the distribution and effectiveness of nanoparticles in real time. Advanced imaging modalities, such as magnetic resonance imaging (MRI), positron emission tomography (PET), and fluorescence imaging, can be integrated with nanoparticle platforms to provide valuable feedback on treatment progress and allow for timely adjustments. Methodological limitations also hinder the advancement of nanoparticle-based therapies. Standardizing the production and characterization of nanoparticles is essential to ensure consistency and reproducibility across studies. The lack of comprehensive preclinical models that accurately mimic the human tumor microenvironment poses a challenge. Developing more sophisticated in vitro and in vivo models will be crucial for testing the efficacy and safety of nanoparticle-based therapies before clinical trials.

### 7.3. Potential of Further Research

Further research in nanoparticle-based therapies holds immense potential. As our understanding of tumor biology and hypoxia mechanisms deepens, new targets and strategies for intervention will emerge. Chemotherapy often requires multiple doses (e.g., six cycles) because the treatment needs to be administered over time to be effective. Thus, it is highly essential to focus on incorporating anti-hypoxic drugs into a system that can provide sustained effects for a longer period. Polyethylene glycol (PEG) has been widely used as a polymeric steric stabilizer, increasing a system’s blood circulation time. It can be incorporated on the liposomal surface to develop stealth liposomes, which are phospholipid bilayer membrane-coated vesicles that can escape detection by the mononuclear phagocyte system, resulting in long-term time system exposure. Current practice focuses on encapsulating the drug into a carrier to develop an effective delivery system. Additionally, this system achieves high drug loading, target efficiency, and activity. Additional focus is needed to develop multifunctional nanoparticles that are capable of delivering multiple therapeutic agents simultaneously or sequentially and could provide more effective treatments by targeting different aspects of tumor hypoxia and resistance. Research into personalized nanoparticle therapies tailored to individual tumors’ genetic and molecular profiles could lead to precise and effective treatments. Combining nanoparticles with other modalities such as immunotherapy, radiation, or gene editing could also open new avenues for synergistic treatments that overcome the limitations of single-agent therapies. While there is no definitive endpoint, nanoparticle technologies’ continuous evolution and integration with other therapeutic approaches suggest a dynamic and rapidly advancing field. The goal is to develop highly effective, safe, and personalized treatments that can be widely adopted in clinical practice.

### 7.4. Future Directions and Evolution of the Field

The future of research in nanoparticle-based therapies for targeting tumor hypoxia is promising. As advancements in nanotechnology, molecular biology, and oncology converge, we can expect significant breakthroughs that will transform cancer treatment. Key areas likely to see substantial progress include the development of intelligent nanoparticles with responsive or programmable drug release mechanisms, integrating diagnostic and therapeutic (theranostic) capabilities, and using artificial intelligence to design and optimize nanoparticle formulations. In the next five to ten years, the standard procedure for treating hypoxic tumors may significantly shift. We may see the routine use of personalized nanoparticle therapies that are tailored to the unique characteristics of each patient’s tumor. Integrating real-time monitoring and feedback systems could enable dynamic adjustments to treatment plans, ensuring maximum efficacy and minimal side effects.

### 7.5. Speculative Viewpoint for the Next Five Years

In five years, it is plausible that nanoparticle-based therapies will have transitioned from being a promising research area to a standard part of cancer treatment protocols, particularly for tumors that exhibit significant hypoxia. Advances in nanotechnology will likely lead to the development of more sophisticated and versatile nanoparticles that can deliver a combination of drugs, genes, or immunomodulators in a controlled and targeted manner. These therapies could be integrated with real-time imaging and monitoring systems to provide a comprehensive treatment solution that adapts to the patient’s evolving needs. Moreover, the field may witness the emergence of regulatory guidelines designed explicitly for nanoparticle-based therapies, facilitating their clinical translation and adoption. Collaborative efforts between researchers, clinicians, and regulatory authorities will be crucial in establishing these guidelines and ensuring that the new therapies are safe and effective.

Overall, the future of nanoparticle-based therapies for targeting tumor hypoxia looks bright. With continued research and development, these innovative treatments have the potential to improve cancer treatment outcomes significantly, offering new hope for patients with hypoxic tumors and contributing to the broader goal of personalized and precision medicine.

## Figures and Tables

**Table 1:** Metabolic reprogramming by hypoxia and possible targets.

Pathways	Enzymes	Effects	Example	References
Glucose transport	GLUT1, GLUT3	Helps transport glucose for tumor cell survival	GLUT1 inhibitor WZB117	[12]
Glycolysis	Aldolase A, HK1, PKM2, and LDHA	Facilitates glycolysis in tumor cells	FX11 inhibits LDHA;pharmacological inhibition of PKM2	[13]
Mitochondrial oxidative metabolism	PDK1 expression	PDK1 regulates PDH, the enzyme responsible for converting pyruvate to acetyl-coenzyme A, which enters the tricarboxylic acid cycle	Dichloroacetate is a potent PDK inhibitor that has been shown to trigger apoptosis in preclinical animals	[14]
Lipid metabolism	Lipin-1	Absorption and lipid droplet accumulation	Numerous FAS inhibitors have demonstrated anticancer efficacy, including cerulenin, C75, orlistat, C93, and GSK837149A	[15]

GLUT1—glucose transporter protein type 1; GLUT3—glucose transporter protein type 3; WZB117—a prototypic anticancer drug; HK1—hexokinase 1; PKM2—pyruvate kinase isozyme M2; LDHA—lactate dehydrogenase A; FX-11—a potent, reversible, and competitive inhibitor of LDHA; PDK1—pyruvate dehydrogenase kinase 1; PDH—pyruvate dehydrogenase; FAS—fatty acid synthase.

**Table 2:** List of different nanoformulations developed to enrich oxygen availability or supply.

Type	Nanoformulation	The Reason Behind the Development	Achievement
Lipid	LN [LN (DOX + ICG)]	Controls the invasiveness and aggressive growth of gliomas	Incorporation of four different functional constituents like targeted delivery to gliomas, rapid release of DOX, image-guided surgery, and inhibition of glioma growth [57]
	LipoMB/CaO_2_	Hypoxia limits the efficiency of traditional PDT	Due to O_2_’s self-sufficient property, LipoMB/CaO_2_ demonstrated dual-stage light-driven PDT [58]
	2,2-azobis [2-(2-imidazoline-2-yl)propane] dihydrochloride (AIPH)-loaded lipid nanoparticles	A hypoxic environment reduces the therapeutic outcomes	Lipid nanoparticles deliver polymerization initiators that generate free radicals in an oxygen-dependent manner [59]
	Photosensitizer-loaded perfluorocarbon nanodroplets	Inadequate oxygen supply during PDT efficacy	Oxygen self-enriching photodynamic therapy accelerated the production of ^1^O_2_ and elevated cytotoxicity [60]
	Liposomal trans-crocetin (L-TC)	A natural product that can help with hypoxia by increasing the diffusion of oxygen in plasma and tissues	Successfully used in phase 2 clinical trial for preoperative hypo-fractionated radiotherapy to localized or locally advanced soft-tissue sarcoma [61,62]
Polymer	Fluorescent platinum complex encapsulated in Quencher-2 doped with PLGA	Increased production of H_2_O_2_ is associated with many cancers that are responsible for oxidative stress	Stimuli-responsive carriers release drugs and O_2_ sustainably [60]
	MnO_2_ NPs encapsulated into PLGA to produce PLGA-MnO_2_ NPs	Hypoxia triggers immunosuppression in TME, resulting in the inhibition of the cytotoxic function of natural killer cells	PLGA-MnO_2_ NPs demonstrated O_2_ production and sustained high O_2_ tension; this nanocomposite reduces hypoxia after penetrating cancer spheroids [63]
	Polyethylene glycol (PEG)-Poly L-lysine (PLL)-Poly lactic-co-glycolic acid (PLGA)-based nanoparticles modified by transferrin-loaded daunorubicin (DNR-Tf-PEG-PLL-PLGA-NPs)	Hypoxia is a critical component of solid tumors and hampers cancer therapy	The downregulation of HIF-1α was observed with the DNR-Tf-NPs; additionally, significant induction of apoptosis was detected by overcoming the hypoxia [64]
Metal	PEG-functionalized hollow Bi_2_Se_3_ nanoparticles (PEG-Bi_2_Se_3_@PFC@O_2_)	Hypoxia-associated resistance to radiotherapy is a big challenge	Hollow nanoparticles carried O_2_ to improve tumor oxygenation: an effective therapeutic outcome; O_2_-loaded perfluorocarbon nanodroplets enhance RT therapy [65].
	PEG-stabilized PFC nanodroplet decorated with TaOx nanoparticles (TaOx@PFC-PEG)	The absorption of radiation energy by tumor cells is less during hypoxic conditions	TaOx@PFC-PEG absorbs X-rays, increasing radiation energy in tumor cells, whereas PFC supplies O_2_ in the TME [66]
	Gold nanoparticle (GNPs)	Hypoxia impairs the therapeutic efficacy of radiotherapy	GNPs combined with radiotherapy improved the antitumor effect in hypoxic tumors [67]

LN—lipid nanoparticles; DOX—doxorubicin; ICG—indocyanine green; LipoMB/CaO_2_—liposome-based nanoparticle containing methylene blue; PDT—photodynamic therapy; PLGA—poly(lactic-co-glycolic acid); NPs—nanoparticles; H_2_O_2_—hydrogen peroxide; MnO_2_—manganese dioxide; TME—tumor microenvironment; PEG—polyethylene glycol; TF—transferrin; DNR—daunorubicin; HIF1α—hypoxia-inducible factor-1α; PFC—perfluorocarbon; TaOx—tantalum oxide.
